# Investigation of the Presence of Glaucoma in Patients with Obstructive Sleep Apnea Syndrome Using and Not Using Continuous Positive Airway Pressure Treatment

**DOI:** 10.4274/tjo.galenos.2018.88614

**Published:** 2019-06-27

**Authors:** Ahmet Abdullayev, Oya Tekeli, Özge Yanık, Turan Acıcan, Banu Gülbay

**Affiliations:** 1 Ankara University Faculty of Medicine, Department of Ophthalmology, Ankara, Turkey; 2 Ankara University, Faculty of Medicine, Department of Chest Diseases, Ankara, Turkey

**Keywords:** Glaucoma, ganglion cell complex, obstructive sleep apnea syndrome, ocular hypertension, optic disc

## Abstract

**Objectives:**

To evaluate the frequency of glaucoma in patients with obstructive sleep apnea syndrome (OSAS) using and not using continuous positive airway pressure treatment.

**Materials and Methods:**

This prospective study included 59 patients diagnosed with OSAS based on the Apnea-Hypopnea Index (AHI). OSAS patients were divided into 3 groups according to their AHI scores: 5-15 was considered mild (19 patients), 16-30 was considered moderate (16 patients), and >30 (24 patients) was considered severe. Twenty-eight (47.5%) of the OSAS patients had been using continuous positive airway pressure treatment. The control group included 19 healthy subjects. Retinal nerve fiber layer and ganglion cell complex (GCC) thickness analyses were performed.

**Results:**

Average GCC thickness in left eyes was significantly lower in the mild OSAS group than in the control group (p=0.013). The GCC was significantly thinner in the inferior and inferonasal sectors of both eyes in the mild OSAS group compared to the control group (p=0.029, p=0.022, p=0.037, and p=0.019 respectively). Minimum GCC thickness in the left eyes of all OSAS groups was significantly lower than in the control group (p<0.05).

**Conclusion:**

In OSAS patients, there may be changes in retinal nerve fiber layer and ganglion cell complex thickness before alterations in the visual field emerge.

## Introduction

Obstructive sleep apnea syndrome (OSAS) is characterized by reduction of air flow or interrupted respiration due to repeated upper respiratory tract blockages during sleep, and is often associated with decreased oxygen saturation.^1^ In the adult population, it is estimated to affect 1.2-2.5% of women and 1-5% of men.^2,3^ Studies have reported higher prevalence of primary open-angle glaucoma (POAG) in OSAS patients as well as higher prevalence of sleep disorders in POAG patients.^4,5^ Several mechanisms have been proposed to explain the development of glaucomatous optic neuropathy in OSAS patients, including dysregulation of optic nerve head blood flow as a result of repeated prolonged apneas, disruption of optic nerve blood flow secondary to arteriosclerosis and arterial blood flow changes, and optic nerve damage induced directly by repeated prolonged hypoxia.

Glaucoma is a progressive optic neuropathy characterized by degeneration of retinal ganglion cells.^6^ It has been shown that 40% of retinal ganglion cell axons may be lost before visual field defects develop in glaucoma patients. In this study, we aimed to evaluate retinal nerve fiber layer (RNFL) and ganglion cell complex (GCC) thickness in OSAS patients.

## Materials and Methods

This prospective study included 59 patients diagnosed with OSAS. The OSAS patients were contacted by phone and invited for ophthalmological examination. The control group consisted of 19 healthy individuals who were fully evaluated to rule out OSAS signs and symptoms. After the study procedures were explained to the participants in full, informed consent forms were obtained. The study was conducted in line with the Declaration of Helsinki. Approval was obtained from the Ethics Committee of Ankara University prior to the initiation of the study (date: 13 October 2014, 16-686-14). The OSAS patients were divided into three groups based on the apnea-hypopnea index (AHI). AHI of 5-15 events/hour was considered mild, 16-30 was considered moderate, and >30 was considered severe OSAS. According to this classification, there were 19 patients (37 eyes) in the mild group, 16 patients (31 eyes) in the moderate group, and 24 patients (47 eyes) in the severe group. The methods used for OSAS treatment in patients were also questioned. Twenty-eight OSAS patients (47.5%) (2 in the mild group, 8 in the moderate group, and 18 in the severe group) were under continuous positive airway pressure (CPAP) treatment.

### Exclusion criteria were:

a) History of intraocular surgery,

b) History of ocular trauma,

c) History of uveitis,

d) Family history of glaucoma,

e) Hypermetropia greater than +4 diopters (D) and/or myopia greater than -5 D; astigmatism exceeding ± 1.00,

f) Presence of retinal disease,

g) History of antiglaucoma medication use at any time in the past,

h) Presence of corneal opacity interfering with optical coherence tomography (OCT) imaging,

i) Previous retinal laser treatment for any reason,

j) Presence of central apnea,

k) Presence of optic neuropathies.

A complete ophthalmological examination was performed on all participants. Iridocorneal angle was analyzed in four quadrants using a gonio lens (Ocular Instruments, Washington, USA). Intraocular pressure (IOP) was measured using Goldmann applanation tonometry. Central corneal thickness (CCT) measurements were determined ultrasonically using a pachymetry device (Ocuscan RXP Alcon, USA). SITA standard 24-2 visual field test (Humphrey Field Analyzer Model 750i, Zeiss, USA) was performed. Tests complying with reliability criteria (less than 20% loss of fixation, 33% false negatives) were included in the study. Automated visual field analyses were performed at least twice on all subjects. After dilatation of the pupil with 1% tropicamide, the fundus was examined.

Optical coherence tomography (Cirrus HD-OCT, Carl Zeiss Meditec, Inc, software version 4.0) was used to evaluate the optic disc and RNFL. The GCC was analyzed using ganglion cell analysis (GCA) software. Cirrus HD-OCT is a spectral domain OCT device with a scanning speed of 27.000 A-scans per second. Measurements were performed using the optic disc cube 200x200 scanning protocol. Optic disc cube is a glaucoma scanning protocol that monitors the optic disc and parapapillary retinal region in a 6x6-mm^2^ area (200x200 data points). Rim area, disc area, and vertical cup/disc ratios were recorded in the disc analysis. RNFL thickness was determined as the average of the whole image and within quadrants. Ganglion cell-inner plexiform layer (GCIPL) was examined using the macular cube 512x128 protocol on the GCA software. Average, minimum, and sectoral (superior, inferior, superonasal, superotemporal, inferonasal, inferotemporal) GCIPL thicknesses were measured in the oval ring around the fovea. Measurements with signal power of 6 and above were used to prevent segmentation errors. Images with movement artifact or signal power lower than 6 were repeated. All measurements were performed prospectively by the same physician (A.A.). Three measurements were taken for each eye and the average values were calculated.

### Statistical Analysis

The data were analyzed using the SPSS for Windows 15 package software. Descriptive statistics were given as mean ± standard deviation for normally distributed variables, median (minimum-maximum) for nonparametric variables, and number of eyes and percentage (%) for nominal variables.

Depending on the distribution of the data, comparisons of means or medians of independent variables were performed using t-test or Mann-Whitney U test. Nominal variables were analyzed using Pearson chi-squared test or Fisher’s Exact test. Regarding the relationships among continuous variables, Spearman correlation test was used for non-normally distributed data and Pearson correlation test was used for normally distributed data. P<0.05 was accepted as the criterion for statistical significance.

In the statistical analyses, right and left eyes were compared between the mild, moderate, and severe OSAS groups and the control group. Left and right eyes were also compared within the groups.

## Results

Fifty-nine patients with OSAS confirmed by polysomnography and a control group consisting of 19 healthy individuals were included in the study. OSAS patients were divided into 3 groups based on AHI values: 19 patients (32.2%, 37 eyes) had mild OSAS (AHI 5-15); 16 patients (27.1%, 31 eyes) had moderate OSAS (AHI 16-30); and 24 patients (40.67%, 47 eyes) had severe OSAS (AHI >30). The OSAS patient group included 34 men (57.6%) and 25 women (42.3%). The control group included 6 men (31.57%) and 13 women (68.42%). There was a statistically significant difference in sex distribution between the groups (p=0.018). Table 1 shows the demographic characteristics in detail.

Three eyes of 2 patients with severe OSAS had ocular hypertension (OHT). However, the automated visual field test and optic nerve analyses were normal in both cases and they were included in statistical analyses. The frequency of OHT was found to be 3.44% in patients with OSAS.

There was no significant difference between the right and left eyes in the mild, moderate, and severe OSAS groups or the control group in terms of IOP, best-corrected visual acuity (BCVA), mean RNFL and optic nerve head parameters, or mean deviation in visual field testing (p>0.05). Compared to the control group, pattern standard deviation (PSD) values were significantly higher in the right eye in the mild OSAS group and in the left eye in the moderate OSAS group (p=0.051 and p=0.033, respectively). The details are given in Table 2.

Average RNFL thickness values in right and left eyes were 91.0±10.3 µm and 89.7±10.3 µm in the mild OSAS group, 93.2±7.01 µm and 89.6±8.5 µm in the moderate OSAS group, 95.5±10.4 and 93.1±8.7 in the severe OSAS group, and 95.2±9.8 and 95±8.6 in the control group. There were no statistically significant differences among the groups (p>0.05) (Table 3). AHI was positively correlated with average RNFL thickness in left eyes in the moderate OSAS group (p=0.010, r=0.620).

Comparison of average GCC thickness values between right and left eyes of the groups showed significantly lower values in the left eyes of the mild OSAS group compared to the control group (p=0.013). Minimum GCC thickness in left eyes was significantly lower in the mild, moderate, and severe OSAS groups compared to the control group (p=0.010, p=0.019 and p=0.004, respectively). The details are given in Table 4. In comparisons of GCC thickness by sector, significantly lower values were observed in the inferior and inferonasal sectors of right and left eyes in the mild OSAS group when compared with the control group (p=0.029, p=0.022, p=0.037 and p=0.019, respectively). Inferonasal GCC thickness was positively correlated with AHI in right eyes in the mild OSAS group (r=0.594, p=0.007). Superonasal GCC thickness was significantly lower in left eyes in the mild OSAS group in comparison with the control group (p=0.011). In addition, superonasal GCC thickness was correlated with AHI in right eyes in the mild OSAS group (r=0.612, p=0.005). Inferior GCC thickness was significantly lower in the right eyes of the severe OSAS group compared to the control group (p=0.049). Details are shown in Table 3.

Twenty-eight OSAS patients (47.5%) (2 in the mild group, 8 in the moderate group, and 18 in the severe group) were under CPAP treatment. There was no statistically significant difference in age, BCVA, CCT, average RNFL, and ONH values between OSAS patients with and without CPAP and the control group. The mean deviation value in left eyes in the non-CPAP group was significantly higher than that of the control group (p=0.054). Mean PSD values in the right eyes of the CPAP and non-CPAP groups were significantly higher than those of the control group (p=0.016 and p=0.014, respectively). The details are provided in Table 4.

In RNFL analysis by quadrant, RNFL in the nasal quadrant of left eyes was significantly thinner in the non-CPAP group than in the control group (p=0.047). Details are provided in Table 5.

Average GCC thickness was significantly lower in right eyes in the CPAP group than in the control group (p=0.021) and in the left eyes of both the CPAP and non-CPAP groups compared to the control group (p=0.008 and p=0.042, respectively). Similarly, minimum GCC thickness was significantly lower in the right eyes in the CPAP group than in the control group (p=0.039) and in the left eyes of both the CPAP and non-CPAP groups than in the control group (p=0.000 and p=0.005, respectively). Table 5 shows the GCC analysis by sector.

## Discussion

A recent cohort study indicated that sleep apnea was not associated with higher risk of glaucoma.^7^ However, previous studies have reported a wide range of glaucoma prevalence, between 2% and 27%.^8^ In our study, 2 of the 59 OSAS patients (3.44%) were diagnosed with OHT.

In OSAS patients, glaucomatous optic neuropathy may develop as a result of severe hypoxia and the subsequent increase in vascular resistance and decreases in perfusion and oxygen saturation.^9^ Although apnea episodes are temporary, the chronic nature of the disease may lead to structural changes in the RNFL. Some studies have reported decreases in mean RNFL thickness in patients with OSAS.^9,10,11,12^ Moreover, a correlation was reported between OSAS severity and RNFL thickness.^9,10^

An RNFL study by Kargi et al.^10^ including 34 OSAS patients and a control group of 20 individuals showed that RNFL thickness was significantly reduced in OSAS patients. Lin et al.^9^ evaluated 105 OSAS patients and 20 control individuals and reported significantly lower mean RNFL thickness in moderate and severe OSAS groups compared to the mild OSAS and control groups. Gutierrez-Diaz et al.^13^ examined 10 OSAS patients diagnosed with normal-tension glaucoma (NTG), 10 OSAS patients without glaucoma, and 10 participants in a control group and found that RNFL values were significantly lower in the NTG and non-NTG OSAS groups than in the control group. Xin et al.^14^ reported significant thinning of the nasal RNFL in the mild, moderate, and severe OSAS groups and of the inferior RNFL in the mild and moderate OSAS groups compared with the control group. Shiba et al.^15^ reported lower nasal RNFL thickness in both the right and left eyes of 124 OSAS patients compared with other quadrants. They also observed a negative correlation between nasal RNFL and AHI in both eyes.#^*#ref15#*#^ Similarly, Casas et al.^16^ compared 50 OSAS patients and 33 healthy individuals and found that nasal RNFL thickness was significantly lower in the OSAS patients. Topcon 3D and Stratus-OCT devices were used in these studies. However, in our study we used a Cirrus HD-OCT device to compare mild, moderate, and severe OSAS patients with a control group and observed no statistically significant difference in mean RNFL thickness (p>0.05). In the moderate OSAS group, a positive correlation was found between AHI and average RNFL thickness in the left eye (r=0.620, p=0.010). However, this correlation was not clinically significant. In terms of quadrants, there was no difference among groups in the superior, temporal, inferior, or nasal quadrants. In regards to the use of CPAP, we found that nasal RNFL was significantly thinner in the left eyes of patients not using CPAP when compared with the control group (p=0.047).

Kergoat et al.^17^ reported that retinal ganglion cells are especially sensitive to abnormal perfusion and reduced oxygen saturation. When we reviewed the relevant literature, we did not find any study investigating the GCC in OSAS patients. Thus, we analyzed changes in the GCC in OSAS patients. Minimum GCC thickness in left eyes was lower than that of the control group in all three OSAS groups. When patients using CPAP (n=28) and those not using CPAP (n=31) were compared with the control group, the average GCC thickness in the right eyes of the CPAP group was found to be significantly thinner than that of the control group (p=0.021). In left eyes, average GCC thickness was lower in the CPAP and non-CPAP groups in comparison with the control group (p=0.008, p=0.042). Minimum GCC thickness in right eyes of the CPAP group and left eyes of the CPAP and non-CPAP groups was thinner compared with the control group (p=0.039, p=0.000, and p=0.005, respectively). In sector analysis, inferior and inferonasal GCC was thinner in the right eyes of the CPAP and non-CPAP groups compared to the control group (p=0.005, 0.022, 0.006, 0.041, respectively). In left eyes, GCC was thinner in the superior, inferotemporal, inferior, and inferonasal sectors in the CPAP group compared to the control group (p=0.033, 0.047, 0.005, 0.010, respectively) and in the superonasal sector in both the CPAP and non-CPAP groups in comparison with the control group (p=0.010 and 0.013, respectively).

Recently, Shinmei et al.^18^ studied IOP changes during nocturnal sleep using a contact lens sensor and reported immediate decrease in IOP during apnea phases. This finding showed that IOP-independent etiology such as the vascular hypothesis may be the mechanism underlying the association between OSAS and glaucoma. Vasodilatation caused by hypoxia and hypercapnia in OSAS patients indirectly disrupts cerebral perfusion and blood flow to the optic nerve by increasing intracranial pressure. This mechanism may explain the RNFL and GCC thinning in OSAS patients. As symptoms are more noticeable in severe and moderate groups, such patients usually visit otorhinolaryngology and pulmonology clinics. Patients in the mild group visit doctors less frequently due to fewer symptoms, which prolongs the duration of untreated illness. The longer untreated duration in mild cases may make retinal ganglion cells more sensitive. This may explain why the results of our study were mostly significant in the mild group.

## Conclusion

Patients with OSAS may be more likely to have OHT or glaucoma. Hence, patients should be monitored thoroughly for glaucoma development and both otolaryngologists and pulmonologists should be informed about this issue. As RNFL and GCC changes may precede visible optic disc and visual field abnormalities in glaucoma, periodic evaluation of RNFL and GCC thickness may have diagnostic value in the early detection of glaucoma in OSAS cases.

## Figures and Tables

**Table 1:**
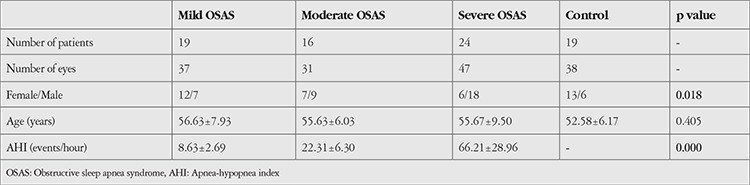
Demographic characteristics of obstructive sleep apnea syndrome patients and the control group

**Table 2:**
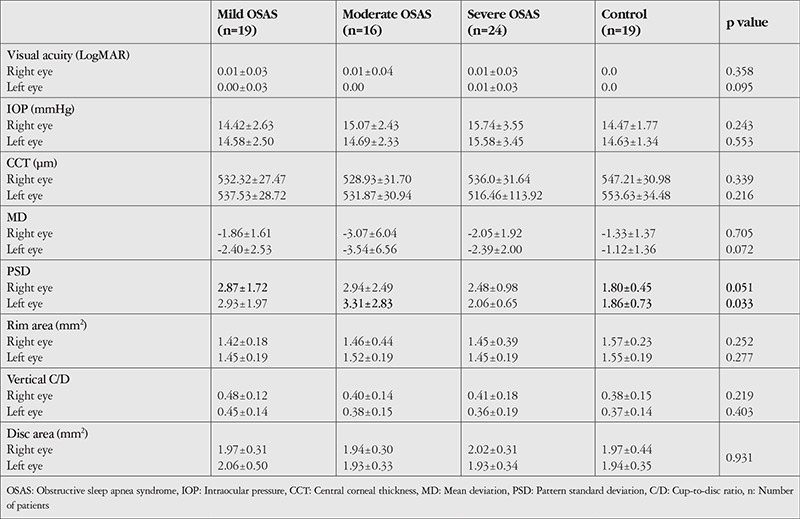
Comparison of visual acuity, intraocular pressure, central corneal thickness, visual field and optic nerve head parameters among groups

**Table 3:**
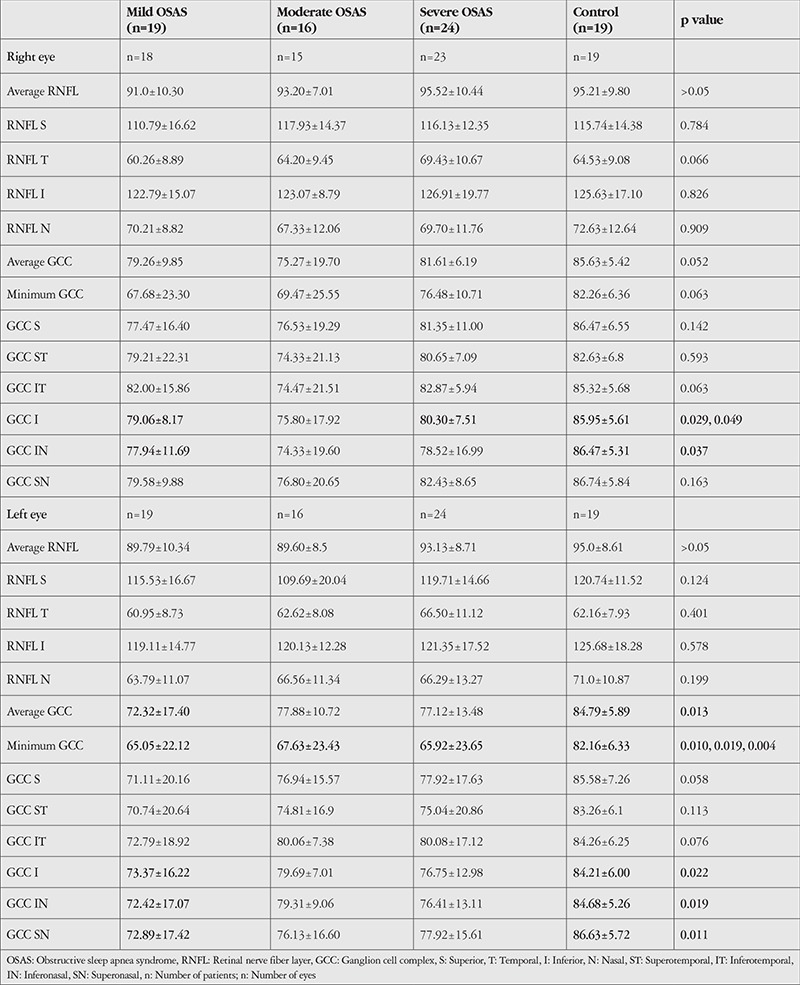
Comparison of retinal nerve fiber layer and ganglion cell complex thickness (μm) among obstructive sleep apnea syndrome groups

**Table 4:**
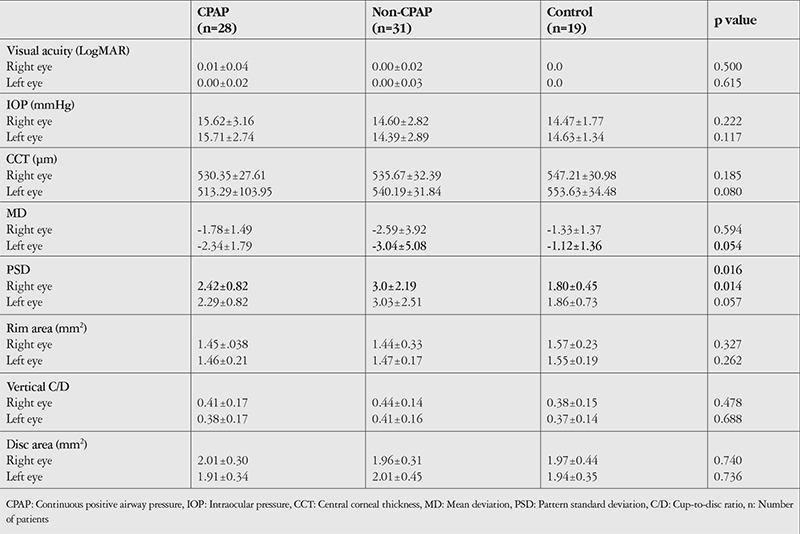
Comparison of patients using CPAP, those not using CPAP, and the control group

**Table 5:**
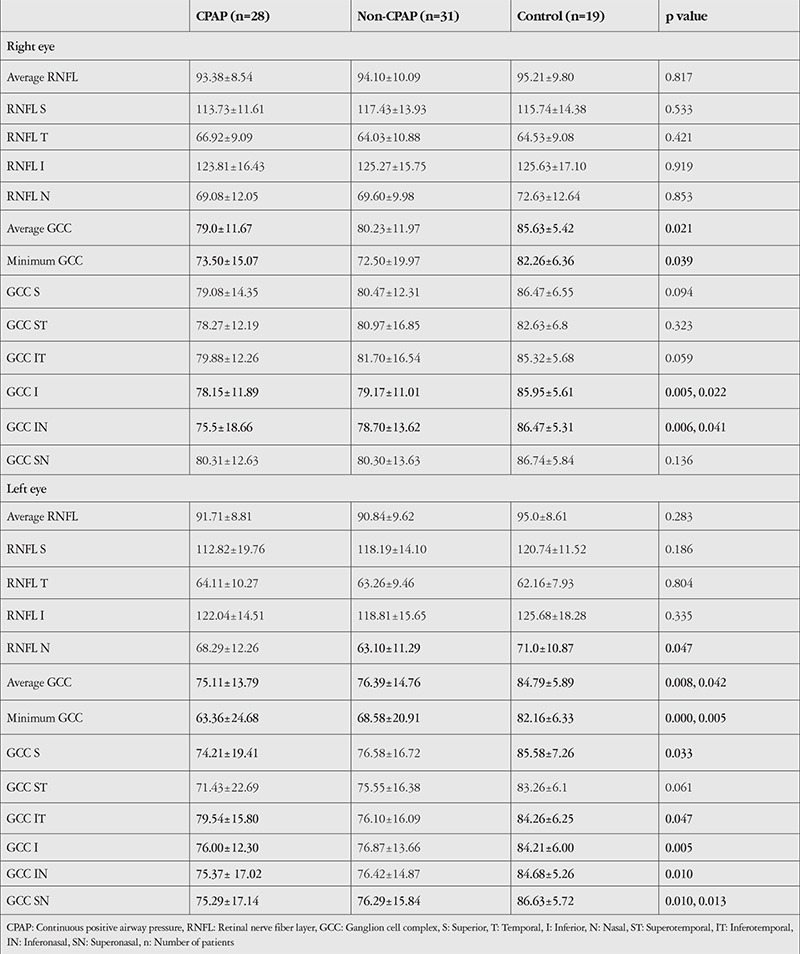
Comparison of RNFL and GCC thickness (μm) in the group using CPAP, the group not using CPAP, and the control group
